# Impact of Dental Trauma on Orthodontic Parameters—A Systematic Review and Meta-Analysis

**DOI:** 10.3390/children10050885

**Published:** 2023-05-15

**Authors:** Mohammad Khursheed Alam, Mohammed Awawdeh, Ali S. Aljhani, Ghada Serhan Alotaib, Huda Abutayyem, Haytham Jamil Alswairki, Mohammad Younis Hajeer

**Affiliations:** 1 Orthodontic Division, Preventive Dentistry Department, College of Dentistry, Jouf University, Sakaka 72345, Saudi Arabia; 2 Department of Dental Research Cell, Saveetha Institute of Medical and Technical Sciences, Saveetha Dental College and Hospitals, Chennai 600077, India; 3 Department of Public Health, Faculty of Allied Health Sciences, Daffodil International University, Dhaka 1207, Bangladesh; 4 Preventive Dental Science Department, College of Dentistry, King Saud bin Abdulaziz University for Health Sciences (KSAU-HS), Riyadh 11426, Saudi Arabia; jhania@mngha.med.sa; 5 King Abdullah International Medical Research Center, Ministry of National Guard Health Affairs, Riyadh, 11481, Saudi Arabia; alotaibigh@mngha.med.sa; 6 Dental Services, King Abdulaziz Medical City, Ministry of the National GuardHealth Affairs, Riyadh 11426, Saudi Arabia; 7 Department of Clinical Sciences, Center of Medical and Bio-Allied Health Sciences Research, College of Dentistry, Ajman University, Ajman P.O. Box 346, United Arab Emirates; h.abutayyem@ajman.ac.ae; 8 School of Dental Sciences, Universiti Sains Malaysia, Kota Bharu 16150, Malaysia; hitham.swerki@gmail.com; 9 Department of Orthodontics, Faculty of Dentistry, University of Damascus, Damascus P.O. Box 16046, Syria; myhajeer@gmail.com

**Keywords:** dental trauma, root resorption, tooth avulsion, orthodontic therapy

## Abstract

Background and objectives: Investigation into the impact of dental trauma on the results of orthodontic treatment is crucial because it can have a major influence on patient care. However, there has not yet been a thorough review or meta-analysis of the available data, which is inconsistent and scant. Therefore, the goal of this systematic review and meta-analysis is to investigate the impact of dental trauma on orthodontic parameters. Search methods and criterion of selection: Major online databases were searched (beginning from the year 2011) for relevant articles using a properly defined search strategy. Analysis protocol: Risk of bias (RoB) and the Cochrane risk of bias tool were utilized for the purposes of bias evaluation within the individual studies and within the review, respectively. Results: Out of the six clinical trials selected, a significant impact of trauma was observed in individuals in all but one paper. Gender predilection varied across studies and could not be conclusively determined. The follow-up period ranged from two months to two years in the trials. The odds ratio (OR) 0.38 [0.19, 0.77] and the risk ratio (RR) 0.52 [0.32, 0.85] indicated that both the odds as well as the relative risk of experiencing dental trauma were lower in the group with negligible impact compared to the group with noticeable impact. Conclusion and further implications: The findings show that dental trauma significantly affects orthodontic parameters, with lower risk and likelihood of suffering dental trauma in the group with negligible impact than in the group with noticeable impact. However, given the substantial heterogeneity among the studies, it is advised to exercise caution when extrapolating the findings to all populations. Registration and protocol: Registration in the PROSPERO database was carried out before initiating the investigation [CRD42023407218].

## 1. Introduction

Dental trauma refers to any injury or damage to the teeth or surrounding tissues caused by physical impact or trauma [1]. It is a common occurrence, especially among children and adolescents, and can range from minor chips and cracks to more severe injuries, such as avulsed or completely knocked-out teeth. It can result from a wide range of causes, including sports injuries, falls, car accidents, and violence [2]. The severity of the trauma depends on various factors, such as the force of the impact, the angle of the impact, and the type of object that caused the trauma [3].

The impact of dental trauma can extend beyond the immediate physical damage to the teeth and surrounding tissues [1]. Dental trauma can also lead to long-term complications such as malocclusion, root resorption, and periodontal disease. These complications can have significant implications for the patient’s overall oral health and well-being, as well as their quality of life [4]. Treatment for dental trauma varies depending on the severity and nature of the injury [5,6]. Minor chips and cracks can often be treated with cosmetic procedures such as bonding or veneers, while more severe injuries may require more extensive treatments such as root canal therapy, dental implants, or orthodontic treatment [7]. In some cases, immediate emergency treatment may be necessary to save the affected teeth [6]. Prevention is key when it comes to dental trauma. Regular dental check-ups can also help to detect and treat any dental problems early before they develop into more serious issues [6].

The different types of dental traumas include enamel fracture, enamel–dentin fracture, crown–root fracture, and root fracture [1]. An enamel fracture is the least severe type of trauma, which involves the breaking of only the enamel layer of the tooth. An enamel–dentin fracture is a more severe form of trauma, which involves the breaking of both the enamel and the underlying dentin layer [2]. A crown–root fracture is a more severe form of trauma that involves the fracture of the tooth crown and extends below the gum line into the root of the tooth. A root fracture is the most severe form of dental trauma, which involves the breaking of the root of the tooth and often results in the loss of the tooth. Dental traumas can occur at any age, but they are more common in children and young adults [3]. The permanent dentition is more commonly affected by dental traumas than the primary dentition, with the upper central incisors being the most commonly affected teeth. The incidence of dental traumas is also higher in males than females. The time of occurrence of dental traumas depends on the type of injury [2]. Enamel fractures and enamel–dentin fractures are commonly seen in children, while crown–root and root fractures are more commonly seen in young adults [4]. Sports injuries, such as those incurred during football, basketball, and soccer, are the most common causes of dental traumas in children, while motor vehicle accidents and physical assaults are common causes in young adults.

The damage caused by dental trauma can vary widely depending on the type and severity of the injury and the location of the trauma [8]. In primary teeth, dental trauma can lead to tooth displacement, avulsion, crown fracture, or root fracture. In mixed dentition, traumatic injuries can also cause tooth displacement, avulsion, crown, or root fracture but may also result in damage to the permanent teeth that are still developing [3]. When a child experiences dental trauma, prompt evaluation by a dental professional is critical [1]. The choice of treatment depends on the type and severity of the injury, as well as the age and stage of dental development of the child. In cases of mild dental trauma, conservative management may be appropriate, such as observation and monitoring for any changes over time. However, in more severe cases of dental trauma, intervention may be necessary. In cases of dental trauma in the primary or mixed dentition, orthodontic or orthopedic treatment may be required to address any resulting malocclusions or misalignments [2]. Orthodontic treatment may involve the use of braces, aligners, or other appliances to correct any tooth movement or misalignment. Orthopedic treatment, on the other hand, aims to address any skeletal discrepancies that may have arisen as a result of dental trauma [6]. The use of orthodontic or orthopedic treatment in cases of dental trauma in the primary or mixed dentition can be beneficial in preventing further damage to the developing permanent teeth. By correcting any misalignments or skeletal discrepancies, orthodontic or orthopedic treatment can also improve the child’s oral function and aesthetics, thereby improving their overall quality of life [6].

Dental trauma can have a significant impact on orthodontic treatment, which involves the correction of dental and skeletal abnormalities through the use of braces, aligners, and other appliances [7,8]. When a patient with dental trauma seeks orthodontic treatment, their dentist or orthodontist must carefully assess the extent of the damage and the potential impact on their orthodontic treatment plan [9]. One of the most common ways in which dental trauma can affect orthodontic treatment is by causing malocclusion or misalignment of the teeth and jaws [10]. This can occur when teeth are chipped, cracked, or knocked out, altering the position of adjacent teeth and disrupting the natural alignment of the bite. In some cases, the impact of the trauma can also cause the teeth to move, leading to further misalignment [11]. Dental trauma can also lead to a variety of complications, which can either arise immediately or manifest later on. Immediate complications may include fractures, dislocations, and soft tissue injuries, while late complications may include root resorption, pulp necrosis, and periodontal damage [10]. The occurrence of these complications is highly dependent on the type of injury sustained, its severity, and the appropriateness of any prior treatments. When orthodontic treatment is initiated, it is important to take into account any history of dental trauma and the possible long-term consequences that may arise [11]. Specifically, the orthodontist must consider the type and severity of the previous injury, as well as the timing and appropriateness of any prior treatment, in order to optimize outcomes for the patient [11].

The impact of dental trauma on orthodontic treatment outcomes is an important topic of research, as it can have significant implications for patient care. However, the current evidence on this topic is inconsistent and limited, with no systematic review or meta-analysis to date. Therefore, the aim of this systematic review and meta-analysis is to investigate the impact of dental trauma on orthodontic parameters. The review examined studies that assessed the impact of dental trauma on orthodontic treatment outcomes. Specifically, the review assessed the effect of dental trauma on orthodontic parameters such as tooth movement, occlusal relationships, and the impact on the patients who were undergoing treatment in the selected studies.

## 2. Materials and Methods

### 2.1. PICOS Strategy

The following PICOS strategy was adopted for our current investigation.

Population (P): The population for this study included individuals who had experienced dental trauma and were undergoing orthodontic treatment.

Intervention (I): The intervention of interest was the impact of dental trauma on orthodontic parameters, including tooth movement, occlusion, and arch development.

Comparison (C): The comparison group for this study were individuals who had not experienced dental trauma and were undergoing similar orthodontic treatment (either using the same modalities or none at all).

Outcome (O): The primary outcome of interest was the impact of dental trauma on orthodontic parameters, as measured by changes in tooth movement, occlusion, arch development, or pain perception. Secondary outcomes included the incidence and severity of dental trauma, as well as the effectiveness of orthodontic treatment in individuals who had experienced dental trauma.

Study Design (S): This study was a systematic review and meta-analysis of clinical trials that examined the impact of dental trauma on orthodontic parameters. The study only included clinical trials published after 2011 to ensure that the most up-to-date evidence was included and subsequently reviewed in the analysis.

By following this PICO strategy, the study was able to answer the research question in a systematic and comprehensive manner, and the results provide important insights into the impact of dental trauma on orthodontic treatment outcomes based on the most recent evidence available. On the basis of this PICOS strategy, the research question formulated was “What is the impact of dental trauma on orthodontic parameters, including tooth movement, occlusion, and how effective is orthodontic treatment in individuals who have experienced dental trauma?”.

### 2.2. Search Protocol

After identification of the relevant MeSH terms, we conducted a search across 4 major databases using Boolean operators. The strategy implemented is given as follows:

- PubMed: ((“Dental Trauma”[Mesh] OR “Tooth Injuries”[Mesh]) AND (“Orthodontics”[Mesh] OR “Malocclusion”[Mesh])) AND (“Clinical Trial”[ptyp] OR “Controlled Clinical Trial”[ptyp] OR “Randomized Controlled Trial”[ptyp]);
- Web of Science: TS = (“Dental Trauma” OR “Tooth Injuries”) AND TS = (“Orthodontics” OR “Malocclusion”) AND PT = (“Clinical Trial” OR “Controlled Clinical Trial” OR “Randomized Controlled Trial”);
- Scopus: TITLE-ABS-KEY(“Dental Trauma” OR “Tooth Injuries”) AND TITLE-ABS-KEY(“Orthodontics” OR “Malocclusion”) AND (DOCTYPE(ar) OR DOCTYPE(re));
- Google Scholar: allintitle:(“Dental Trauma” OR “Tooth Injuries”) AND allintitle:(“Orthodontics” OR “Malocclusion”) AND (“Clinical Trial” OR “Controlled Clinical Trial” OR “Randomized Controlled Trial”).

### 2.3. Registration Protocol

**Figure 1:**
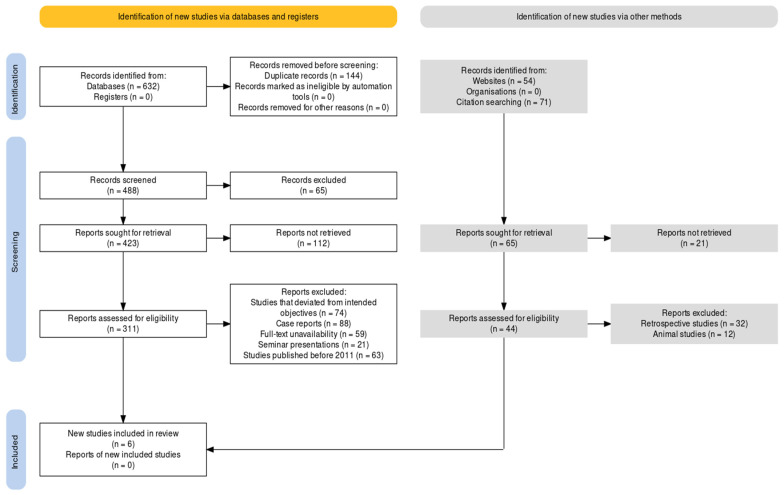
Paper selection protocol using the PRISMA framework.

The PRISMA guidelines [12] were used to ensure the systematic and transparent reporting of the study and to facilitate the assessment of the quality of the study (Figure 1). The protocol included a detailed description of the research question, search strategy, inclusion and exclusion criteria, data extraction process, and statistical analysis plan. Prior registration in the PROSPERO registration network was carried out to increase transparency and reduce the risk of bias in the study. The protocol included the registration number assigned by PROSPERO [CRD42023407218], which allowed for easy identification and tracking of the study. The registration protocol also included a description of the study design, including the rationale for conducting a systematic review and meta-analysis, the selection of studies, and the statistical methods used to analyze the data. The protocol also included a discussion of the potential limitations of the study and the steps taken to address them. This protocol was designed to ensure a rigorous and transparent approach to the study and to increase the confidence in the validity and reliability of the results.

### 2.4. Inclusion and Exclusion Criterion

The inclusion and exclusion criteria for this systematic review and meta-analysis were developed to ensure that only high-quality studies were included in the analysis. Inclusion criteria were studies that investigated the impact of dental trauma on orthodontic parameters, clinical trials published after 2011, studies that included a comparison group of individuals who did not experience dental trauma but underwent similar orthodontic treatment, studies that reported on outcomes related to changes in tooth movement, occlusion, and arch development, studies that reported on the incidence and severity of dental trauma, and studies that reported on the effectiveness of orthodontic treatment in individuals who have experienced dental trauma.

On the other hand, exclusion criteria were studies that investigated the impact of dental trauma on orthodontic treatment outcomes but were not clinical trials, studies that were published before 2011, studies that did not include a comparison group of individuals who did not experience dental trauma but underwent similar orthodontic treatment, studies that did not report on outcomes related to changes in tooth movement, occlusion, and arch development, studies that did not report on the incidence and severity of dental trauma, and studies that did not report on the effectiveness of orthodontic treatment in individuals who have experienced dental trauma.

By applying these inclusion and exclusion criteria, the systematic review was able to ensure that only relevant and high-quality studies were included in the analysis, which in turn would allow for a more accurate and reliable investigation into the impact of dental trauma on orthodontic parameters.

### 2.5. Study Selection Protocol

The data selection protocol for this systematic review and meta-analysis involved a rigorous and systematic approach to study selection and data extraction. Two reviewers were brought in to independently assess each study for inclusion in the analysis based on the predefined inclusion and exclusion criteria. In case of any disagreement between the two reviewers regarding the inclusion or exclusion of a study, a third reviewer was consulted to help resolve the query. The data selection process was documented in detail, and all selected studies were obtained in full text for further analysis. Using a predefined data extraction form, the same two reviewers independently extracted data from each study. The extracted data was then cross-checked for accuracy and completeness by the two reviewers. Any discrepancies in the extracted data were resolved through discussion between the two reviewers or involving a third reviewer if necessary. Finally, the final data set was compiled and used for statistical analysis. By following this data selection protocol, the study aimed to ensure a rigorous and systematic approach to study selection and data extraction, minimizing the risk of bias and ensuring the reliability of the findings.

### 2.6. Bias Assessment

**Figure 2:**
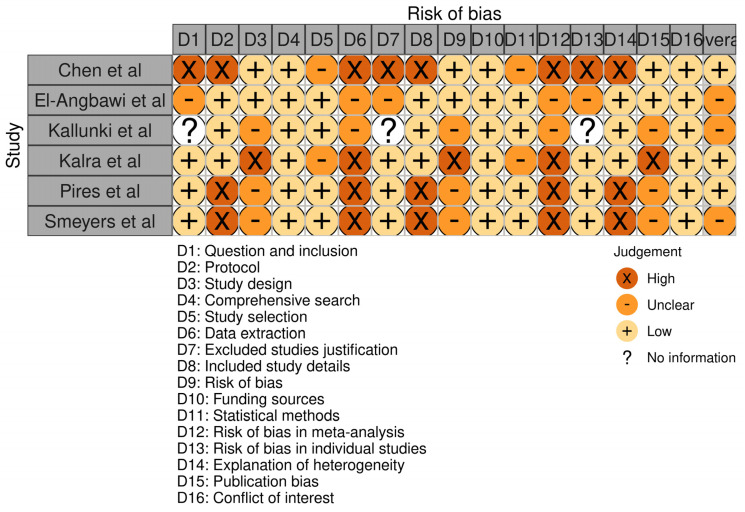
Evaluation of bias in the selected investigations using the RoB−2 tool.

**Figure 3:**
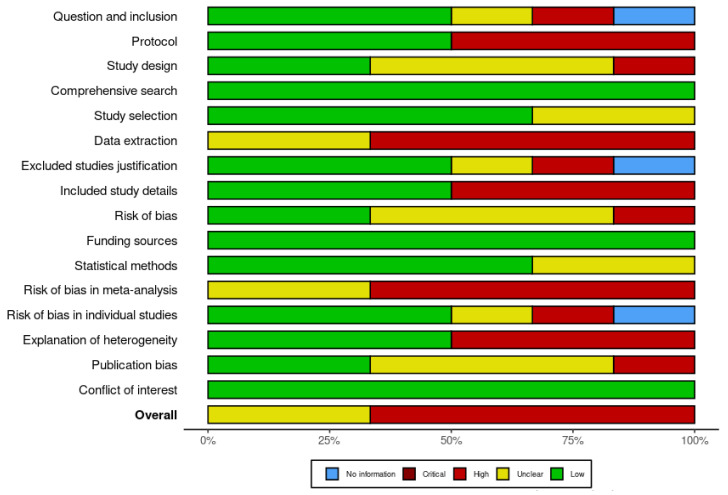
Risk of bias assessment within the review using the Cochrane risk of bias tool.

Each included paper was assessed for risk of bias using the RoB-2 tool (Figure 2) [13]. This involved evaluating the study across five major domains. Each domain was evaluated as having a low, high, or some concern risk of bias. The overall risk of bias for each study was then determined based on the evaluation of each domain. After this, the review itself was assessed for bias using the Cochrane bias assessment checklist [13]. This involved evaluating the review across several points, which are represented in Figure 3. Each point was evaluated as having a low, high, or unclear risk of bias. The overall risk of bias for the review was then determined based on the evaluation of each point. Using these tools to assess bias, the study aimed to ensure that the included RCTs were evaluated for their risk of bias and that the review itself was evaluated for potential sources of bias. This approach helps to increase the rigor and reliability of the study’s findings by identifying potential sources of bias and taking steps to minimize their impact on the results.

### 2.7. Statistical Protocol Employed

The RevMan 5 software (version 5.4.1) was used for the statistical analysis of our investigation. Both odds ratio (OR) and risk ratio (RR) were analyzed, assuming a 95% confidence interval (CI) and random effects (RE) model. The software was utilized to conduct a meta-analysis of the selected trials, which involved pooling data from multiple studies to estimate the overall effect size. The software was used to input the data from the selected trials and to calculate the OR and RR values for each trial. The software then calculated the overall effect size and provided a forest plot to visualize the results. The software also calculated the heterogeneity Tau^2^, Chi^2^, df, I^2^, and Z values, which were used to assess the degree of heterogeneity and the significance of the overall effect. The use of this tool allowed for a comprehensive and systematic analysis of the data, which provided important insights into the relationship between dental trauma and orthodontic parameters. The results of the meta-analysis can inform future research and clinical practice in this area and can help to improve patient outcomes by identifying the most effective treatment approaches.

## 3. Results

As represented in Figure 2, the risk of bias in the included studies was evaluated using the RoB-2 tool across various domains. The study by Chen et al. [14] had a low risk of bias in most domains except for concerns related to the excluded studies. The study by El-Angbawi et al. [15] had some concerns regarding the risk of bias in some domains, and the risk of bias was unclear in others. The study by Kallunki et al. [16] had no information regarding the risk of bias in some domains and some concerns in others. The study by Kalra et al. [17] had a low risk of bias in some domains and a high risk of bias in others, particularly with respect to funding sources. The study by Pires et al. [18] had a low risk of bias in some domains and a high risk of bias in others, particularly with respect to data extraction and statistical methods. The study by Smeyers et al. [19] had a low risk of bias in some domains and a high risk of bias in others, particularly with respect to publication bias. In this manner, the present review and meta-analysis showed that there was a variation in the risk of bias across the included studies. While some studies had a low risk of bias in most domains, others had a high risk of bias in one or more domains. The findings of this study should be interpreted with caution, given the variability in the risk of bias across the included studies. The authors suggest that future studies should adhere to rigorous research methodologies to minimize the risk of bias and increase the reliability of the findings.

The search strategy for this systematic review and meta-analysis involved searching four databases, including PubMed, Web of Science, Scopus, and Google Scholar. The initial search yielded 632 articles. After removing duplicates, 488 articles remained. These articles were then screened based on their titles and abstracts. Based on the inclusion and exclusion criteria, 65 articles were excluded, leaving 423 articles for full-text review. These articles were then assessed for eligibility based on the predetermined inclusion and exclusion criteria. Articles were excluded if they did not meet the criteria, were not randomized control trials (RCTs), or were published prior to 2011. After applying these criteria, 54 articles were excluded, leaving a total of 311 articles for further evaluation.

At the end of this search protocol, a total of six RCTs were selected for inclusion in the investigation [14,15,16,17,18,19]. Table 1 represents the demographic variables pertaining to these included studies, whereas Table 2 provides with the technical description of their methodologies and the assessments that were observed in them respectively. One study was published in 2011 [14], one in 2015 [18], and the remaining four studies were published after 2019 [15,16,17,19]. The search strategy and screening process were carried out in a systematic and thorough manner to ensure that only the most relevant and up-to-date studies were included in the final meta-analysis. The selection of only RCTs published after 2011 helped to ensure that the most recent and reliable evidence was included in the analysis.

Out of these six clinical trials, a significant impact of trauma was observed in individuals in all but one paper [15]. Males were found to be more susceptible to dental trauma than females in two of the trials [14,17], whereas in a couple of other trials, no significant correlation could be deduced [18,19]. In the remaining two studies [15,16], the predilection remained unspecified. The follow-up period ranged from two months [17] all the way to two years [16] in the studies that were selected.

**Figure 4:**
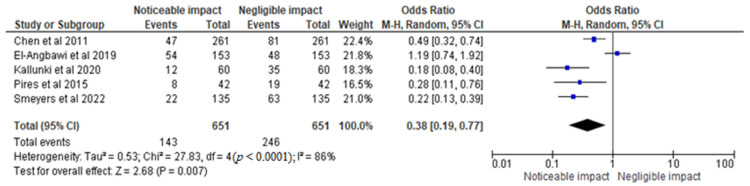
Incidence of dental trauma and its impact on orthodontic parameters in the total sample size of the selected trials represented in terms of the OR [14,15,16,18,19].

For the meta-analysis, the study by Kalra et al. [17] was not considered since the authors did not report the specific percentage/number of people who had experienced dental trauma at any point in time. The rest of the five trials underwent further meta-analysis. The statistical analysis of the incidence of dental trauma and its impact on orthodontic parameters was conducted using data from selected RCTs. The results were displayed in a graph that compared the OR of 0.38 [0.19, 0.77] for negligible impact versus noticeable impact (Figure 4), assuming a 95% CI and RE model. The heterogeneity value Tau^2^ was found to be 0.53, with a Chi^2^ of 27.83 and df of 4 (*p* < 0.0001), indicating significant heterogeneity. The I^2^ value of 86% suggests a high degree of variability in the results across the RCTs. The test for overall effect yielded a Z value of 2.68 (*p* = 0.007), indicating a statistically significant difference between the two groups. Based on the analysis, it can be concluded that dental trauma has a noticeable impact on orthodontic parameters. The odds ratio of 0.38 [0.19, 0.77] indicates that the odds of experiencing a noticeable impact are 62% lower in the group with negligible impact compared to the group with a noticeable impact.

**Figure 5:**
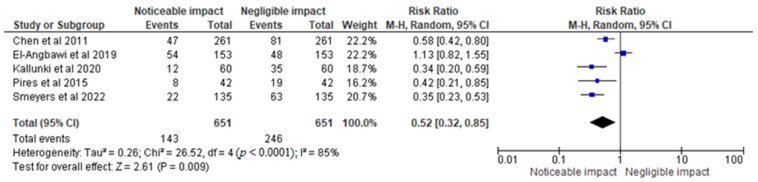
Incidence of dental trauma and its impact on orthodontic parameters in the total sample size of the selected trials represented in percentage terms of the RR [14,15,16,18,19].

In Figure 5, the results were represented in percentage terms of the RR of 0.52 [0.32, 0.85], assuming a 95% CI and RE model. The heterogeneity Tau^2^ was found to be 0.26, with a Chi^2^ of 26.52 and df of 4 (*p* < 0.0001), indicating significant heterogeneity. The I^2^ value of 85% suggests a high degree of variability in the results across the trials. The test for overall effect yielded a Z value of 2.61 (*p* = 0.009), indicating a statistically significant difference between the groups. Based on the analysis, it can be concluded that dental trauma has a significant impact on orthodontic parameters. The risk ratio of 0.52 [0.32, 0.85] indicates that the risk of experiencing dental trauma is 48% lower in the group with negligible impact compared to the group with noticeable impact. However, the significant heterogeneity suggests that the results may not be generalizable to all populations, and further research is needed to confirm these findings. Overall, the statistical analysis provides important insights into the relationship between dental trauma and orthodontic parameters and can inform future research and clinical practice in this area.

## 4. Discussion

The analysis of the selected RCTs revealed that dental trauma has a significant impact on orthodontic parameters. The study found that out of the six clinical trials analyzed, a significant impact of trauma was observed in individuals in all but one paper. This suggests that dental trauma has a noticeable impact on orthodontic parameters. The odds ratio and risk ratio both indicate that the odds and risk of experiencing a noticeable impact are significantly higher in individuals who have suffered dental trauma. However, the significant heterogeneity in the results suggests that the findings may not be generalizable to all populations, and further research is needed to confirm these findings. The I² value of 85–86% suggests a high degree of variability in the results across the RCTs. This variability could be due to differences in the populations, methodology, and other factors. Therefore, future studies should aim to address these limitations to obtain more reliable results. All in all, this study provides important insights into the relationship between dental trauma and orthodontic parameters. The findings can inform future research and clinical practice in this area. Dental practitioners and researchers can use these findings to develop effective strategies for preventing and managing dental trauma, which can help improve the quality of life for individuals who have experienced this type of injury.

This study identified and synthesized the findings from several studies that have investigated the relationship between dental trauma and orthodontic parameters using different orthodontic appliances in a rather unique manner since selecting studies with varying interventions often generates heterogeneity. However, we believed this was important as different types of orthodontic appliances are used in clinical practice, and it is essential to understand the impact of dental trauma on orthodontic parameters in patients treated with different appliances. The present study included studies that used various orthodontic appliances such as Bionators and headgear, fixed appliances, mouthguards, and brackets. Despite the use of different appliances, the findings of the included studies showed that dental trauma can have a significant impact on orthodontic parameters. For instance, Chen et al. [14] reported a significant impact of dental trauma on orthodontic parameters in patients treated with Bionator and headgear. Similarly, Kallunki et al. [16] found a significant impact of dental trauma on orthodontic parameters in patients treated with headgear. The study by Smeyers et al. [19], which included patients treated with both fixed and removable appliances, also found a significant impact of dental trauma on orthodontic parameters, culminating in root resorption. The study by El-Angbawi et al. [15] did not find a significant impact of dental trauma on orthodontic parameters in patients treated with fixed appliances, while the study by Pires et al. [18] reported a significant impact on oral mucosa in patients treated with brackets.

Dental trauma can have a significant impact on orthodontic treatment, but with careful planning and a comprehensive approach, many patients are able to achieve successful outcomes and restore their oral health and function. Orthodontic treatment recommendations for individuals with a history of dental trauma vary not only across clinical contexts [20] but also across dental professional groups [21]. In one study on the impact of dental trauma as perceived by clinicians, respondents acknowledged that in cases of tooth ankylosis, it was impossible to move the tooth orthodontically. The literature also contains extensive documentation of this response [22,23]. The mineralized root surface (cementum or dentin) will fuse with the alveolar bone after a serious injury to the periodontal membrane (such as intrusive luxation or replantation after tooth avulsion). As a consequence, the root structure will gradually be replaced by bone (replacement resorption) [24]. The movement of teeth during orthodontic treatment is not feasible without a periodontal ligament [22]. Surprisingly, in that survey, approximately one in six general dentists were unaware of this. In addition to these reactions, which are not usually connected to tooth ankylosis [24], external cervical resorption and apical root resorption are frequently cited as adverse events in trauma situations [25].

Before beginning orthodontic therapy, there is a high risk of further root breakdown in the apical area if there is apical root resorption [26]. It is still unknown what causes this response in the first place. Frequent orthodontic treatment complication is a condition known as apical root resorption, which occurs when the apical region of the root is damaged due to pressure placed on it during tooth movement [27]. Pulp canal obliteration, also known as calcific metamorphosis, can develop as a result of dental trauma. It has been observed that juvenile teeth with extrusive and lateral luxation injuries experience this condition the most frequently [28,29]. Pulp necrosis and apical pathology are described in the literature as findings connected to the orthodontic movement of teeth impacted by this condition [30]. Progressive obliteration suggests that the pulpal blood flow is being reduced [31]. Compared to traumatized teeth without or with partial pulp obliteration, teeth with complete pulp obliteration are more susceptible to pulpal complications during orthodontic intrusion [32]. Additionally, noteworthy was the frequent mention of root resorption as a potential adverse event in our analysis, despite the fact that the literature does not support this finding [32,33].

After receiving orthodontic treatment, it is advised to follow up with dental trauma patients by having regular radiographic examinations and pulp vitality tests performed at regular intervals [7,8,34]. Furthermore, the majority of research participants agreed that routine radiographic examinations are the best way to manage traumatized teeth that have undergone endodontic treatment. This result is consistent with recommendations made by a study by Owtad et al. [34], which recommended radiographic monitoring by taking radiographs of root-filled teeth before therapy starts and repeating them six months later.

In comparison, 44% of participants in a study felt that treating traumatized teeth with endodontic treatment should be conducted in the same manner as treating non-traumatized teeth [35]. This observation was in accordance with a paper mentioned in the literature [8]. This can be ascribed to the finding that there is no appreciable difference in the root resorption between vital teeth exposed to the same orthodontic forces and teeth that have undergone root canal therapy [8,24].

To address the issues discussed in this review, orthodontic treatment plans for patients with dental trauma often involve a combination of restorative and corrective treatments. Restorative treatments such as fillings, crowns, and dental implants may be used to repair damaged teeth and restore their natural shape and function. Orthodontic treatments such as braces, aligners, and appliances may then be used to correct any misalignments and ensure that the teeth and jaws are properly aligned. In cases where the dental trauma is more severe, additional treatments such as oral surgery may be necessary to address any skeletal abnormalities or damage to the jawbone. It is important for patients with dental trauma to work closely with their dentist or orthodontist to develop an individualized treatment plan that considers their unique needs and concerns. The prognosis of a dental trauma case will deteriorate over time if there is a protracted delay in the replantation or unfavorable storage circumstances [36]. This is a result of changes that take place in the periodontal ligament and pulp, changes that will determine whether the avulsed tooth is saved or lost [36]. The type of treatment needed will depend on a variety of clinical factors: the tooth’s extraoral period, the periodontal ligament’s condition, a preservation medium, and the extent of root development [35]. The severity of the trauma and the extent of the damage also determine the course of treatment that is needed. For minor injuries, such as a small chip or crack, the tooth may be repaired with a filling or dental bonding [36]. In cases where there is more extensive damage to the tooth or surrounding structures, such as a broken or displaced tooth, a root canal or tooth extraction may be necessary [36]. Trauma to the tooth can also lead to other long-term complications, such as root resorption, which is the breakdown of the root of the tooth. This can lead to tooth mobility and eventual tooth loss if left untreated [36]. Additionally, trauma to the tooth can cause changes in the tooth’s position and alignment, which can lead to bite problems and the need for orthodontic treatment [36].

Despite the significant findings of this study, there are several limitations that should be considered. Firstly, the search strategy was limited to four databases and did not include a hand search of reference lists, potentially leading to the exclusion of relevant studies. Secondly, the inclusion criteria were restricted to RCTs published after 2011, which may have excluded relevant studies conducted prior to this time period. Additionally, only studies published in English were included, which may have excluded studies conducted in other languages. Furthermore, the small number of RCTs included in the final analysis may limit the generalizability of the findings. Additionally, the heterogeneity among the studies was high, which suggests that the results may not be applicable to all populations. It is also important to note that the follow-up periods of the included studies varied, with some studies having shorter follow-up periods than others, which may have affected the results. Moreover, the exclusion of one study due to insufficient data may have impacted the accuracy of the meta-analysis. It is also important to acknowledge that the results of this study were limited to orthodontic patients and may not be applicable to the general population. Finally, it is important to consider the potential for publication bias, as studies with significant results may be more likely to be published. Overall, while the findings of this study provide valuable insights into the relationship between dental trauma and orthodontic parameters, further research is needed to confirm these findings and address these limitations.

## 5. Conclusions

This systematic review and meta-analysis provide important insights into the impact of dental trauma on orthodontic parameters. The results indicate that dental trauma has a noticeable impact on orthodontic parameters, with lower risk and odds of experiencing dental trauma in the group with negligible impact compared to the group with a noticeable impact. However, the significant heterogeneity across the studies suggests that caution should be taken when generalizing the results to all populations. Furthermore, the limited number of studies included in the analysis and the small sample sizes of some studies are significant limitations. Despite these limitations, the findings of this study can inform future research and clinical practice in this area, emphasizing the importance of minimizing the impact of dental trauma on orthodontic outcomes.

## Figures and Tables

**Table 1:** Demographic characteristics of the papers evaluated under the review.

Study ID	Year	Scenario Pertaining to Trauma Assessment	Sample Size (*n*)	Age Range (in Years)	Gender Ratio (Male: Female)
Chen et al. [14]	2011	Orthodontic appliance usage	261	9.7 (mean)	145:116
El-Angbawi et al. [15]	2019	Root resorption	153	≥12	48:105
Kallunki et al. [16]	2020	Orthodontic appliance usage	60	8–10	Unspecified
Kalra et al. [17]	2021	Sports	24	12–14.5	14:10
Pires et al. [18]	2015	Orthodontic appliance usage	42	16.7 (mean)	20:22
Smeyers et al. [19]	2022	Root length change	135	<18	Unspecified

**Table 2:** Variables assessed with respect to the incidence of midline diastema in the papers under review.

Study ID	Year	Orthodontic Appliance Employed	Incidence of Trauma in Sample Size (Percentage)	Gender Predilection to Trauma	Oral Region Affected by Trauma	Impact of Trauma on Orthodontic Parameters	Follow-Up Period
Chen et al. [14]	2011	Bionator and headgear	25	Significantly higher in males compared to females	Incisors (both central and lateral)	Significant impact observed	>6 months
El-Angbawi et al. [15]	2019	Fixed appliances	16.4	Unspecified	Root resorption	No significant impact observed	9 months
Kallunki et al. [16]	2020	Headgear	18	Unspecified	Incisors (both central and lateral)	Significant impact observed	2 years
Kalra et al. [17]	2021	Mouthguard	Unspecified	Higher in males compared to females	Unspecified	Significant impact observed	2 months
Pires et al. [18]	2015	Brackets	20	No significant difference observed	Oral mucosa	Significant impact observed	5 months
Smeyers et al. [19]	2022	Both fixed and removable appliances	40	No significant difference observed	Incisors (both central and lateral)	Significant impact observed (culminating in root resorption)	6 months

## Data Availability

All data are available within the manuscript.
